# Gender-based roles, psychosocial variation, and power relations during delivery and postnatal care: a qualitative case study in rural Ethiopia

**DOI:** 10.3389/fgwh.2023.1155064

**Published:** 2023-10-23

**Authors:** Ketema Shibeshi, Yohannes Lemu, Lakew Gebretsadik, Abebe Gebretsadik, Sudhakar Morankar

**Affiliations:** ^1^ Department of Health, Behavior and Society, Jimma University, Jimma, Ethiopia; ^2^ Department of Public Health, Dire Dawa University, Dire Dawa, Ethiopia

**Keywords:** gender, delivery, postnatal care, roles, knowledge, belief, power relation

## Abstract

**Introduction:**

The World Health Organization (WHO) strongly encouraged men to support women in receiving maternal healthcare. However, especially in developing countries, maternal healthcare has traditionally been viewed as an issue in women, with men making little or no contribution, even though sexuality and children are shared products. The study aims to understand how gender-based roles, psychosocial variation, and power relations are related to child delivery and postnatal care (PNC) services.

**Methods:**

The study was conducted in three rural districts of Oromia regional state, Jimma Zone, Ethiopia. An in-depth interview and focus group discussion were held with carefully chosen health professionals, health extension workers, community health development armies, and religious leaders. The data was collected, translated, and transcribed by experienced men and women qualitative researchers. For data analysis, ATLAS.ti version 9 was used. The data were coded and categorized concerning delivery and PNC service utilization. Independent and shared gender-based roles were identified as a means to improve maternal healthcare service delivery.

**Results:**

The result obtained three categories, namely, gender-based roles, psychosocial variation, and power relations. Men can persuade pregnant women to use delivery services and PNC. The place of delivery is determined by the levels of gender-based power relations at the household level, but women are usually the last decision-makers. The belief of the community that giving birth in a health facility makes women look clean and neat, as opposed to home delivery, increases their intention to use maternal healthcare services.

**Discussion:**

The study contributes that **t**he role of a man as a husband is crucial in mobilizing others to carry pregnant women to health facilities, contributing to early intervention during labor. The decision-making capacity of women has improved over time, with men accepting their right to make decisions about their health and fetuses. Home delivery and men not being present during delivery are perceived as signs of backwardness, whereas giving birth in health institutes is seen as a sign of modernization and the rights of women.

## 1. Introduction

Gender is defined as what society believes about the appropriate roles, duties, rights, and responsibilities of people and their attitudes, values, relative power, accepted behaviors, and opportunities based on their sex (1). It is one of the common social determinants of maternal health that includes various ideas, basically concerning how women and men interact and the nature of their relationships (2).

Various declarations and initiatives strongly support gender influences on the utilization of maternal healthcare services. Among others, the most common impetus for the initiative was the 1994 International Conference on Population and Development (ICPD) held in Cairo, Egypt (3), which mentioned that men should be involved in the utilization of various maternal health services (4–7). It was supported by the 1995 conference held on World Women's Day in Beijing, China (8). The Addis Ababa declaration revealed the concept of the involvement of men in maternal healthcare (9). Moreover, in 2015 the World Health Organization (WHO) declared that there was low quality evidence of male involvement in maternal healthcare services, and strongly recommended that men should be involved in maternal and child healthcare (10). Although various works of literature described gender-related roles as having a significant influence on maternal healthcare, limitations have widely been observed in the mechanisms of gender integration in maternal healthcare (3, 11–13).

Gender-based power relations play a key role and are one of the determinants of maternal healthcare access and utilization (14, 15). Health outcomes of women seeking maternal healthcare are largely determined by their autonomy (15–17). However, the WHO recommendation considered that the involvement of men should be promoted in maternal and child healthcare, provided that they respect, promote, and facilitate the choices of women and their autonomy in decision-making (18). Moreover, men are considered the gatekeepers and decision-makers to prompt access to maternal and newborn healthcare (MNH) services both at household and community levels (10). The research findings in Afar and Kefa, Ethiopia, revealed the role of gender as follows: men are dominant decision-makers for maternal healthcare and do not allow women to attend meetings held with the health development armies owing to the fear that they might know their rights about maternal healthcare (19).

Gender-based psychosocial components such as knowledge and belief related to maternal healthcare greatly contributed to the modification of the behavior of men and women regarding the utilization of maternal healthcare services. A study in Indonesia suggested that men should have maternal healthcare knowledge to improve its utilization (20). The process of childbirth was only the burden of women as a study in Malawi stated that the husbands do not know about childbirth (21). Literature showed that addressing gender inequity in maternal healthcare service utilization is difficult when dealing only with women (11). Unless men and women are considered as part of the cause and the solution for the low utilization of maternal healthcare, it is hard to successfully challenge and transform gender norms to improve the desired outcome of maternal healthcare services (12). A qualitative study of African-American parents in the USA mentioned that there is equal investment and interest in having a child and that the required responsibility of having the child is willingly shared between the two parents (22). Therefore, understanding the contribution of gender and integration in maternal healthcare will help ease the burden on women during maternal healthcare (23).

Studies conducted in developing countries including Africa concluded that greater male involvement has the potential to improve maternal and child health conditions (24). Husbands failing to notice the involvement of men is a key predicament in maternal healthcare service utilization, whereas as women reported that the lack of involvement of their husbands could hinder their use of delivery and postnatal care (PNC) services (25). On the other hand, male involvement would be reduced if it was perceived as a rule, emanating from the government, which also stigmatized women who did not have a husband (21). Generally, the role of men is complex and multidisciplinary, including but not limited to intervening to assist their wives in maternal health, including giving birth in the health institutes, and some acknowledge reducing workload, particularly in harsh environments (24). This is because the role of gender at household and community levels is believed to improve the intention of women to utilize delivery services (26).

However, emerging evidence and program experience indicate that engaging men during the time of childbirth and PNC could have a considerable health benefit for women and newborn babies in low- and middle-income countries (27). A study on maternal and child health from Zones (middle administrative body) to the Federal level with various maternal healthcare stakeholders in Ethiopia identified that men have a role in doing physically intensive tasks, whereas women are more engaged in child care, household, and health promotion tasks (28).

Many of the studies did not address gender-based subjective experiences of maternal healthcare; rather, they emphasized the observable behavior of men and women to determine the involvement of men in fulfilling gender equity to improve maternal and child health outcomes (11). To enhance the entrenched gender-sensitive maternal healthcare service delivery, subjective experiences of both men and women such as knowledge, belief, and attitudes must be considered in determining their intention on the utilization of maternal healthcare services. Therefore, the current study explored gender-based norms, psychosocial variations, power relations, and community social supports that contribute to childbirth and PNC services. Oftentimes, most research findings did not consider the reasons why men attended childbirth or saved money for emergency transportation for example, while counting these activities as the involvement of men in maternal healthcare services. In addition to all the gender dimensions, understanding the motivational and driving forces of the involvement of men in maternal healthcare has an enormous contribution to addressing gender inequity that is a burden to maternal and child health (29).

## 2. Materials and methods

### 2.1. Setting

The study was conducted in three rural districts of the Jimma Zone, which is located in the southwest part of Ethiopia. The rural districts are far from the city of the Jimma Zone. There are limited infrastructures such as roads, communication, transportation, and health facilities. The health facilities in all selected districts are not fairly accessible to the community. In all the selected districts according to the Central Statistical Agency of Ethiopia, the total population was estimated to be 180,000–270,000 in 2016 (**30**). The predominant ethnic group is Oromo, and the religion is Muslim.

### 2.2. Study design

This study used a qualitative case study design to explore maternal healthcare services in the dimensions of gender-based roles, psychosocial variations, and power relations during delivery and PNC services.

### 2.3. Participants

The participants included are the Women's Health Development Army (WDA) and the Men’s Health Development Army (MDA), which are connectors of health facilities and communities and the preferred sources of health communicators to the mothers to prepare for birth and related complications (31). The other participants included religious leaders (RL), health extension professionals (HEP), and primary healthcare unit directors (PHCUD) (Table 1).

**Table 1:** Sociodemographic characteristics of participants in the rural Jimma Zone of Ethiopia.

Participant type	Number	Mean age	Sex		Years of involvement		Marital status	Educational status		No. of children	
			M	F	Min	Max		Min	Max	Lower	Higher
Women’s Health Development Army	97	30		F	3	8	Married	No education	Grade 7	3	6
Men’s Development Army	90	45	M		2	6	Married	No education	Grade 10	2	7
Religious leaders	6	45	M		4	10	Married	Grade 3	Grade 9	2	6
Primary healthcare unit directors	6	30	M	F	1	3	Married	Diploma	Degree	2	5
Midwifery nurses	6	28	M	F	1	4	Married	Diploma	Degree	1	3
Health extension workers	6	27		F	2	5	Married	Diploma	Degree	1	3

M, male; F, female; Min, minimum; Max, maximum.

### 2.4. Sampling

Purposive sampling was used to select the study participants, both men and women, based on their role in the community. The selection relied on the consideration of their capacity to understand the gender-based roles of the community, psychosocial variation levels, power relations, and social support about delivery and PNC services. Moreover, their significant role in the community, such as providing healthcare support, counseling, and acting as a leader, had been given great value (31).

### 2.5. Data sources

The data were obtained from the Innovative Maternal and Child Health in Africa (IMCHA) project, which is part of the safe motherhood initiatives aimed at preventing maternal and child morbidity and mortality. The principal investigator was from the Department of Health Behavior and Society at Jimma University; however, all the researchers joined at the beginning of the project. The project was conducted in collaboration with the University of Ottawa, Canada. The data were collected from three rural districts: Gomma, Seka Chekorsa, and Kersa, which are not adjacent to the Oromia regional state, Jimma zone, Ethiopia.

### 2.6. Data collection methods

A focus group discussion (FGD) was held with the WDA and the MDA, whereas in-depth interviews were carried out with religious leaders, health extension professionals, PHCUD, and midwifery nurses.

### 2.7. Data collection tools

The study used semi-structured in-depth interviews and FGD guides to explore data from the selected participants. The data collection process was held using skilled qualitative data collectors, who had been recruited and took a 5-day theoretical and field practice training. The interviews and FGD guides were checked after the pretest was conducted other than the actual study areas, and an indispensable modification was made to ensure they were well-suited to the local sociocultural context.

### 2.8. Data collection approach

At a convenient time and location, data were collected separately from each of the participants. A total of 32 in-depth interviews and FGDs from three districts and six community groups took place, of which there were 12 FGDs and 20 in-depth interviews. The FGDs were with MDA and WDA, whereas the in-depth interviews were with religious leaders, health extension workers, midwifery nurses, and PHCUD. Saturation of data was the reason for limiting the number of interviews and discussions with the participants. Both in-depth interviews and FGDs were held for an average of 75–120 min, respectively, with an average of 90 min. The respondents were selected purposefully from each community group except health extension professionals, midwives, and PHCUD due to their limited numbers in the district health facilities. This study is part of the baseline data to compare with the change in the end-line data after the intervention of the information, education, and communication (IEC) for the selected participants aimed to enhance the utilization of maternal healthcare services and the upgrading of the maternal waiting area with essential equipment.

### 2.9. Data analysis

The data collectors translated and transcribed the collected data soon after data collection. The ATLAS.ti version 9, a computer-assisted qualitative analysis statistical software, was used to analyze the data. The entire FGDs and in-depth interviews were exported to ATLAS.ti, and a new project was created. Initially, the data were coded by two coders based on the perceived gender-based descriptions of the participants. The codes with similar ideas were merged and formed categories such as gender-based roles, knowledge, belief, and power relations on maternal healthcare services during childbirth and PNC. Descriptive methods were the coding strategies. The analysis was performed to explore the descriptions of different health professionals and segments of the community about childbirth and postnatal healthcare services.

### 2.10. Trustworthiness

To ensure the rigor of this research, we critically considered an ample number of criteria. First, adequate data from various community and health professionals were collected to produce significant findings. Second, plenty of time was spent in the field to obtain trustworthy results. Third, we identified theoretical goals that should alienate the context of the study subjects. Fourth, we followed a strict procedure when conducting interviews, writing field notes, and analyzing data. Moreover, the four basic measures of qualitative research trustworthiness were considered during data collection and analysis. The first was credibility: to achieve this, data were collected by experienced teams of data collectors; data saturation was considered; data were translated and transcribed timely; and an extensive field engagement was held. Furthermore, detailed descriptions were provided for the categories of gender perspective and supportive quotations, which added value to the content. In addition, multiple data collection methods from various groups of participants (multivocality) were used to construct a multifaceted, more complicated, and more credible picture of the result. The second was dependability: to ensure this, peer debriefing and audit trials were performed during data collection, translation, and transcription. The third was transferability: the data were collected from a resource-limited setting and multiple data triangulation, hence giving the findings a methodological and practical contribution to the body of knowledge of gender-based maternal healthcare since there was limited evidence. The fourth was conformability: to ensure this, the process of data analysis (interpretation) was strictly grounded by the data to keep away from the preferences and viewpoints of the researchers.

### 2.11. Ethical considerations

Potential respondents were given verbal information about the aim of the study and invited to participate with their willing consent. Consent was obtained verbally by the data collectors just before the time of the interviews. Field notes and tape recordings were made to remember the natural setting and enable proper transcription. To ensure confidentiality and anonymity, we did not mention the names of the participants; rather, the verbatim responses of the participants were taken as a quotation to support the description of the category. The FGD was undertaken at the residences of the participants at a convenient time before they started their daily activities, whereas the in-depth interview was conducted at the offices of the participants. A limited amount of money for transportation purposes was reimbursed for FGD participants. Ethical approval was obtained from Jimma University Research Ethics Institutional Review Boards (JUEIRB) and the University of Ottawa.

## 3. Results

### 3.1. Participant profiles

A total of 95 female and 97 male participants from the community were involved in the FGD, whereas four religious leaders, four health extension workers (HEWs), four PHCUDs, and four midwifery nurses were involved in the in-depth interview. The mean age was 41 years old for the male participants and 35 years for the female participants. Their educational status ranges from no formal education to a university degree. The number of children they have ranges from no child to 10 children (Table 1).

### 3.2. Description of gender-based categories during delivery and PNC

This qualitative study identified three thematic categories, namely, gender-based roles during delivery and postdelivery, gender psychosocial variations, and gender-based power relations. These categories focused on the roles of men and women in facilitating and preventing the utilization of maternal healthcare services. Gender-based knowledge and beliefs also played a role in these categories, either supporting or hindering service utilization. The study also examined how individual and collective decision-making influences childbirth and PNC services in health facilities (Table 2).

**Table 2:** Coding guide applied in the second stage of analysis.

Code category	Description	Number of codes identified	Thematic categories
Gender perception of institutional delivery and PNC	What women and men know and believe about delivery and postnatal services	75	Gender-based psychosocial variation to delivery and PNC
Social support	The support men and women in the household and community provided to pregnant women to give birth in the health institutes and use PNC	26	Gender-based roles to delivery and PNC
Mechanisms of men support	The various activities of men's performed support to the pregnant women to facilitate institutional delivery	54	
Men mental involvement	The thought of men to support pregnant women in institutional delivery	18	
Structural ally to maternal health	The various activities of women's performed support to the pregnant women to facilitate institutional delivery	5	
Gender relation	The discussion held between men and pregnant women on the utilization of maternal healthcare services	7	Gender-based power relation to delivery and PNC
Collective decision-making	The decision made by the community members of men and women to support pregnant women in institutional delivery	11	
Individual decision-making	The decision made by the community members of men and women to support pregnant women in institutional delivery	17	

### 3.3. Gender-based roles during delivery and PNC

Gender-based maternal healthcare roles embrace an array of genders about delivery and postpartum care as an individual and collective role. It is the social expectation of the appropriate role and behavior of men and women during and after childbirth. Commonly, men and women are concerned about better health outcomes for pregnant women and their newborns.

#### 3.3.1. Healthcare-seeking practices of pregnant women

The role of pregnant women in labor and delivery is paradoxical. Those who seek healthcare in health institutes are reached before labor signs begin, whereas those who stay at home until labor starts prefer to give birth at home if there are no complications. Depending on the condition, they may go to a health facility.

There are women who come at the initiation of their false labor and stay here [at the health facility] for one or two weeks up to their delivery time …… the others come when labor has failed at home. (PHCUD, Kersa District)

They [pregnant women] come to the health center just when the labor starts; before that, they prefer to give birth at home. So, giving birth at a health center is considered the second option. (Husband, Seka District)

There are pregnant women who are refusing birth at the health center. (Religious leader, Seka District)

Those pregnant women who decided to give birth at home do not tell Shane [one to five women local network] members. Rather, they simply keep quitting, and the entire family says that she [the pregnant woman] is fine. (WDA, Gomma District)

#### 3.3.2. Women assisting their fellow women with domestic chores

The collective gender norms of women facilitate institutional delivery by involving various activities such as escorting pregnant women to health institutes, performing household chores, and providing care for children left at home during childbirth care.

Some women have gone with her [laboring women]; they prepared a coffee ceremony and drank it while she was in health care. She has been morally supported until her return home. She didn't suffer from anything while she was at the health center. (Husband, Gomma District)

Those women who left at house prepared the place where we take rest after returning home, as well as anything else at home; for example, preparing coffee, cooking food, and making all things at home ready. (Woman who gave birth, Kersa District)

The other women who are still at home clean the house well and stay with her children and manage all the necessary things at home. (WDA, Seka District)

#### 3.3.3. Men providing means of transportation to health facilities

Men, including husbands, play a crucial role in facilitating transportation, purchasing food and medicine, and escorting laboring women to health facilities. They intend to save the lives of the woman and newborn and to relieve family responsibility in case of the death of a pregnant woman during delivery at home.

The husband advises her [pregnant wife] … because he is afraid of the problems and expenses that may come with childbirth. The husband has the feeling whatever maternal problems happen; it will affect or hurt his family first. Therefore, he needs to take care by taking the pregnant women to health facilities. (Husband, Gomma District)

Men at the household level play a crucial role in informing community leaders about labor signs and mobilizing community members if ambulance services are unavailable to transport laboring women to health facilities on main roads.

What is expected from the husband is only asking for assistance [the community], then they inform each other and easily come for help. (RL, Gomma and Seka Districts)

The husband tells to EDIR (local traditional support system using regular money collection) leader, so the leader will mobilize the public. Then the people immediately carry the pregnant woman to health care using traditional hand-crafted beds. (MDA, Gomma District) 

…. we all [male community members] are with her once the labor begins. We go with her till her arrival at the health center. We attentively stay there to follow her condition and whether she is being referred or not. We never go to our daily work that day. Every member of MDA, the neighbor, and the community at large should attend her. We do whatever is needed; we carry if there is no ambulance. (Husband, Gomma District)

#### 3.3.4. Healthcare practices of women during PNC

PNC in women is often limited, with a focus on child care like breastfeeding. This is due to cultural restrictions that prevent women from moving outside the home after delivery. Family members, including husbands and neighbors, perform most domestic activities. Three categories of perceptions of women of PNC attendance are identified: those who stay at home for 40–45 days without receiving care, those who do not visit health facilities but are visited by HEW, and those who live near health facilities who visit on the 7th-day postdelivery.

The reason the women do not use PNC is because they think themselves and their baby are healthy and decided to come for immunization only. (Midwifery nurse, Gomma District)

Women who understand the importance of the PNC service could use it. (PHCUD, Seka District)

The mother and the baby will be free of all the problems, as we [HEW] have seen during PNC. (HEW, Seka District)

There is a traditional belief that obligated women who gave birth not to be outside for 40–45 days after delivery. So, we [midwifery] recommend health extension workers conduct PNC-2 visits. (Midwifery nurse, Gomma District)

#### 3.3.5. Husband responsible for filling the household gap

The role of men during the postdelivery period was associated with fulfilling all the necessary foodstuff for the women who gave birth and the family as a whole, which was a continuation of the predelivery preparation. In general, the role of the husband is to fill all the gaps that existed during the postnatal period to maintain the dignity of the family.

He [the husband] is also concerned about the food she will use after delivery and the clothes that she wears. (WDA, Gomma District)

… So that the husbands make ready and clean the bedroom where she will stay (after delivery). (Husband, Seka District)

He (the husband) has a fear that friends and neighbors will laugh at him for the lack of things at home like clothing as well as anything else while visiting them after his wife gives birth. Therefore, he supports his wife very much. (Woman who gave birth, Seka District)

… if they [parents] fail to get a person who supports her in doing things while she is at rest (post delivery), the husband is the one who does that. (WDA, Gomma District)

### 3.4. Gender-based power relations during delivery

Gender-based power relations can be categorized into three main types: shared decision-making by parents, final decision-making by the pregnant woman, and decision-making by men. Parents, including close relatives, share the decision-making process. The place of delivery is determined by the woman, despite prior discussions. Men make decisions on behalf of women in stressful situations or based on their interests. However, controversial issues arise regarding the final decision-maker.

#### 3.4.1. Shared gender-based power relations in choosing the place of delivery

Commonly, both men and women partners held discussions and reached agreements about the place of delivery in a health institution. In addition to the shared decision-making roles of the parents, families of both parents and health extension professionals were involved in the decision-making, particularly in giving birth in the health institutes. However, since the husband and wife were living together initially, they held discussions and reached a consensus on the place of delivery before moving on to the next steps of the decision-making process.

For deciding the place of delivery, she [the pregnant woman] talks with her husband and family, so that she can do as she prefers. Even so, he [pregnant woman's husband] is the one who let her go to the right health facility as soon as she felt pain at this time. Therefore, husbands are not denying women the right to go to a health facility while they face labor; rather, they take her to the facility. (Woman who gave birth, Seka District)

Her [the pregnant woman's] parents, his [husband] parents, and HEWs also participate in the decision to encourage the pregnant woman to go to a health center [at the time of labor]. (Husband, Kersa District)

Even if she has the ability to decide where to give birth, the neighbor also decides for the pregnant woman … … the husband is also behind her and protects her from any harm. (WDA, Gomma District)

Even though this was a rare case, men did not accept the decision to give birth in a health facility. Therefore, women could move on to informing their neighbors or the one-to-five women network to receive assistance at the health facility.

Generally, she [pregnant woman] decides with her husband; if he denies her the right to give birth at a health facility, she again decides with her neighbor and asks them to take her to a health facility. (WDA, Seka District)

#### 3.4.2. Decision-making power of women during delivery service

Woman discussants and religious leaders described that if the discussion between partners failed and did not reach an agreement, pregnant women would have been the last decision-makers to select the place of delivery. They rationalize her decision as, *after all, it was her life that might be in danger; therefore, nothing impeded the woman from deciding on institutional delivery*.

The pregnant woman herself has to make the final decision. Currently, pregnant women are starting to make the decision by themselves. After all, it is about her health and life. (RL, Seka District)

Pregnant women by themselves make the decision [for institutional delivery]. The husbands accept what the pregnant woman says …  At this time, they have been using the health center for their own interests. (RL, Gomma District)

As she preferred to give birth at a health facility, nothing hindered her from doing so since it was her right. Regarding her decision, she starts talking and discussing with her husband by saying, “I want to go [to] a health facility for delivery,” so she says to him [husband] “Either you take me to a health facility or I will go by myself” while she faces labor. Then, if he said no, she [the pregnant woman] would need to go to the health facilities; she would go by herself and give birth there at the health facility*.* (WDA, Seka District)

#### 3.4.3. Decision-making power of men during delivery service

Even if the cases were very few, men (husbands) have made decisions alone in the form of motivation and encouragement of the pregnant women about the place of delivery. There were tendencies of women to respect his decision. On the other hand, since the woman is in a stressful and tension-filled situation at the time of labor, men could have decided on her behalf to carry her into the health institutions without requesting her consent. And in other situations, he denied the preference of the pregnant woman regarding the health institution for delivery.

The one who makes decisions is the husband. The woman herself can also decide, but the final decision will be made by the husband, who gets respect and is implemented. However, the husband's decision is just to motivate and encourage her to attend the health center. (RL, Kersa District)

There are husbands who bring their wives to the maternity waiting area without the consent of the women. (Midwifery nurse, Gomma District)

Sometimes mothers [pregnant women] cannot do anything alone. For instance, if she requests that her husband bring her to the health center, he might not bring her. (PHCUD, Seka District)

There are still some pregnant women who say, “That is the decision of my husband, and if he says that you [the women] have to give birth here at home and don’t go to a health facility, she will give birth there at home by his will. Therefore, she is obeying what her husband ordered and not her own interests. (Woman participants, Gomma and Seka Districts)

### 3.5. Gender-based psychosocial variations about delivery and PNC

The study reveals that the knowledge and beliefs of both men and women about delivery and PNC services have shown positive change, while some knowledge gaps and negative beliefs have been observed regarding maternal healthcare services.

#### 3.5.1. Women know the importance of institutional delivery

Historically, women faced morbidity and mortality due to a lack of awareness about maternal health services and their accessibility. However, most women now understand the benefits of health institutions and the negative effects of home delivery. Fear of bleeding and the need to save lives are the most common reasons for the health concerns of women.

… They (pregnant women) say that ‘there is no bleeding if we give birth in a health center.’ (PHCUD, Kersa District)

Mostly, they prefer to give birth at a health center. Due to the risk of giving birth at home and the safety and care provided by professionals at a health center, they prefer a health center. (MDA, Kersa District)

They (pregnant women) know the risks related to delivering at home and the benefits of delivering at health facilities. (Midwifery nurse, Seka District)

… the reason is that, first, there is excessive bleeding during delivery. So; in order to prevent this; they prefer health care. (MDA, Gomma District)

The reason why they [pregnant women] are not giving birth at home is; that they are getting advice. So, the concern for their health makes them attend health care during delivery. (MDA, Gomma district)

According to the perception of health professionals, the vast majority of pregnant women know the difference between giving birth at home and in health institutes. Therefore, they have an improved intention to attend the maternal waiting home, even before the true labor sign has started. Sometimes without waiting for anyone else's assistance, even her husband can find her in the maternal waiting area of a health institute. Most of them were at the health facility during delivery; they would not have been at home.

There are pregnant women who come at the time of the initiation of their false labor and stay here [health facilities] for one or two weeks up to their delivery time. This is due to the awareness they have …. (PHCUD, Seka District)

After knowing the benefits of health services, pregnant women run to a health facility for delivery as soon as they face labor without waiting for anyone to take them. (WDA, Kersa District)

They [pregnant women] know the risks related to delivering at home and the benefits of delivering at health facilities. (Midwifery nurse, Seka District)

The community perceives home delivery as a backward culture, despite significant knowledge improvements in the health institute about the importance of giving birth.

Currently, giving birth at home is considered a **traditional or backward culture**. When she gives birth at home, there are many challenges, like the overflow of blood, and the deaths of the child and mother. Delivering at the health center saves her life and the newborn, gets good care, and the delivery system is safe. (MDA, Kersa District)

#### 3.5.2. Men (husbands) blamed by the community for in-home delivery

Husbands felt ashamed when pregnant wives gave birth at home, fearing community members would insult them for their irresponsibility and failure to provide advice in health institutions, especially if they knew the women had given birth at home.

… [Community] ask her [women] where she delivered [gave birth]; if she delivered at home, we [the community members] insult the husband; why he did this? All in all, giving birth at home is considered a backward culture, and people are becoming familiar with giving birth at a health center. (MDA, Seka District)

#### 3.5.3. Knowledge of women regarding maternal healthcare

Similarly, as participants expressed, the knowledge of women about institutional delivery was associated with evaluating the health facility delivery services in terms of the cleanliness and neatness of the women after completing the delivery services, with minimal blood loss, unlike home delivery. Another discussant agreed, saying that only a knowledge deficit prohibited women from giving birth in the health institutes.

They are giving birth at a health facility by considering no loss of blood at delivery and delivering neatly, unlike previous times where there was high bleeding up to the extent that it spoiled the odor of the women. (WDA, Kersa District)

They [pregnant women] prefer to give birth here at the health post, as they don't have anywhere else to go for delivery. The reason for preference is to save one life from death. (WDA, Kersa District)

Except for a lack of knowledge, nothing hinders women from using health services, as they can all access them. (WDA, Seka District)

After knowing the benefits of health services, pregnant women run to health facilities for delivery as soon as they face labor without waiting for anyone to take them. (WDA, Seka District)

… most of the time, mothers prefer to give birth at home due to a lack of awareness, but those who do have awareness prefer a health center. (MDA, Seka District)

#### 3.5.4. An improved knowledge of men during labor and delivery

Despite improvements in knowledge about maternal healthcare, men still have less awareness than women. The improved knowledge of men focused not only on advising and instructing the women to visit health centers but also on escorting them during labor and delivery. The participants explained that, unlike the previous time, men have improved knowledge of the importance of institutional delivery and prefer their wives to give birth in a health facility rather than at home unless there is a financial problem.

Since the husbands are afraid of problems occurring during pregnancy, they prefer that their wives give birth at a health center. In cases where the pregnant woman needs to give birth at home, the husband takes her to a health center when the labor comes. The husbands never influence or force their wives to give birth at home. (RL, Gomma District)

Husbands are not denying women giving birth at health facilities unless they are poor. (WDA, Gomma District)

#### 3.5.5. Knowledge level of parents determines their decision

With a low level of knowledge about institutional delivery, some men blame themselves after they have observed safe institutional delivery, while his pregnant wife was forced to come to the health facility for delivery services with the support of a neighbor after he refused to let her give birth in the health facility.

Even if her husband blamed his neighbors for that time—who insisted he carry them to the health facility—he acknowledged them a lot after the pregnant woman came to the health facility and attended safe delivery, he said that, “I had lost my child and my wife at that time due to my poor knowledge.” (HEW, Gomma District)

Another religious leader supported the understanding of the community in general terms by denying the existence of any counter-advocacy for institutional delivery in the community.

There are no individuals who are advocating against maternal health services. (RL, Kersa District)

However, men could prevent pregnant women from attending the network group meetings of women due to the priority given to their personal interest, which is associated with the lack of knowledge of women regarding their rights and fear of their husbands. Despite the network, this gives the pregnant women an opportunity to share their interest and intention about the importance of health facility delivery.

The one thing is that, due to a lack of awareness, they may not have adequate knowledge and do not have education; in addition, they may fear their husband. (Midwifery nurse, Gomma District)

Male farmers want to keep their wives at home to prepare food and warm the house while they return from their work. This can hinder their participation. (PHCUD, Seka Chekorsa District)

There are husbands who say, “Stay at home, until I [the husband] am coming from my work.” We [women] are even having such kinds of husbands. (WDA, Kersa District)

#### 3.5.6. Knowledge related to institutional delivery does not guarantee PNC

According to the explanations of the diversified group of participants, the level of knowledge of pregnant women has improved; they have a clear awareness of maternal healthcare services and maternal health problems associated with home delivery.

More than anyone, pregnant mothers know the effect of giving birth at home. (PHCUD, Gomma District)

Because they are following different media, they [pregnant women] can tell you many things, because they know the problems they may face. (HEW: Seka District)

All women in the locality have equal knowledge as health extension workers. (WDA, Gomma District)

The levels of understanding of pregnant women have been increasing. (RL, Kersa District)

Commonly, due to cultural concerns, most of the women who attended institutional delivery could not come for a postnatal checkup. In addition to the culture, the knowledge gap is another contributing factor.

All those who gave birth will not come for this service [PNC] on the third and seventh days, according to the new guidelines, even if we encourage them to come. (PHCUD, Kersa District)

#### 3.5.7. Gender-based beliefs identified during delivery and PNC services

Gender-based beliefs can either facilitate or hinder the utilization of maternal healthcare services by women and the engagement of men in such services. Culturally, the beliefs of women during delivery, such as exposing their bodies to external people, are considered taboo and prevent them from using services. However, beliefs during delivery, such as giving birth in a health facility to prevent bleeding, save lives, and maintain cleanliness, can facilitate service utilization. In PNC, culture-related gender beliefs that prevent women from moving outside the home before 45 days are often mentioned as hindering factors for PNC services.

##### 3.5.7.1. Beliefs of women in institutional delivery make them clean

The study indicates a shift in the beliefs of women from home birth to health institutions, leading to an unexpected increase in institutional delivery due to a positive transformation of belief in maternal healthcare services.

In previous times, many of them gave birth at home. But now, at least more than a hundred women give birth in health facilities every month. (PHCUD, Seka District)

They [pregnant women] are leaving their homes for the maternal waiting area before anyone sees them. We [men] simply find them at the [maternal] waiting area. They go to the [maternal] waiting area before getting weak. (MDA, Gomma District)

Home delivery is often perceived as harmful to women, but hygiene and cleanliness can change this perception. Returning women home like “brides” improves their perception of cleanliness, bleeding absence, and lack of unpleasant odors, leading to increased delivery service utilization.

Mostly, they prefer to give birth at a health center, due to the risk of giving birth at home and the safety and care given by professionals at health centers. (MDA, Kersa District)

We [the community] didn’t even know how to stop it well, but they stopped the bleeding properly, and that woman would return home. In this current season, both the bride and the woman who gave birth to the health institutes are similar. (WDA, Gomma District)

Nowadays, women do not compare their shame [due to exposing their private parts] with the benefit of facility delivery, like preventing their lives and their children's lives from death and any harm. Therefore, they are giving birth at a health facility by considering no loss of blood at delivery and delivering neatly, unlike previous home delivery times, where there was high bleeding up to the extent of spoiling the odor of the women. (HEW, Kersa District)

##### 3.5.7.2. Shift of professional gender choice during delivery

According to the explanations of religious leaders, unlike previous experiences, pregnant women in the present prefer to give birth in health institutes; previously, “women who gave birth at home were an indication of strength,” which is used to cure the stigma of weakness given by the societies.

At this time, they [the pregnant women] have been using the health center for their own interests. Previously [before the expansion of health facilities], giving birth at a health center was considered shameful. Strong women were the ones who gave birth at home. Therefore, they didn't give birth at the health center. (RL, Gomma District)

There was a tendency to be shy about giving birth in the health institute, especially if the health professionals were male, which was associated with a cultural belief where exposure to others was not allowed. However, things have now changed, as long as they understood the benefit of saving the lives of women and newborns, preventing bleeding, and becoming clean and neat after delivery. Therefore, most pregnant women have shown an intense interest in giving birth in health institutions, regardless of the gender of the health professionals.

There is an improvement [in the health institution] gradually, and nowadays’ women are not comparing shame with the benefit of facility delivery, like preventing their and their children's lives from death and any harm. Therefore, they are giving birth at a health facility by considering no loss of blood at delivery and delivering neatly, unlike previous times where there was high bleeding up to the extent of spoiling the odor of the women. (WDA, Kersa and Gomma Districts)

##### 3.5.7.3. Belief in the positive outcomes of prior malpractices are consistently repeated

Some men have the belief that the negative health outcomes that occurred to the women and the newborns are a result of health facility delivery vis-à-vis all the previous home deliveries which did not create any health problems for the women or the newborns.

Even if she came and delivered at a health center, if there is a problem with the mother or child, they will probably consider this a mistake done by health professionals or as if they murdered his wife or child. They will try to justify this by stating that she had given these numbers [of children born at home and healthy children] at her home, but now this is the result of her coming to the health center. (HEW, Gomma District)

Men often show reluctance to maternal healthcare of their wives due to strong religious beliefs. They believe that “Allah,” rather than modern medicine, protects the health of women and children. This leads to a loss of interest in attending delivery services, a challenge mentioned by a PHCUD.

… Then I asked whether his wife attended delivery service for the current pregnancy, and he [the pregnant woman's husband] replied she didn't. When I asked the reason, he related the issue with religion, and he said protection of one's health was not due to getting modern medication; Allah is the one who protects us from any diseases. … We faced such similar challenges. (PHCUD, Seka District)

##### 3.5.7.4. Societal rule influences the individual behavior

The community has a rule that men should support women giving birth in health institutes, as they could be responsible for undesired health outcomes if pregnant women give birth at home. This structural protection, along with the positive beliefs of men about maternal healthcare services, helps them change their beliefs toward institutional delivery services.

He [the husband] will be responsible for any harm that will happen to that [pregnant] woman if she does not use the [maternal] service during pregnancy and delivery. (Woman who gave birth, Kersa District)

… The husband will be the one responsible and accountable for her if anything happens to that pregnant woman, including after her death if it happens. (Woman who gave birth, Gomma District)

There are rules and regulations taken as legal ways established at shane and gare [locally assigned names for women's association] levels to punish such kinds of crime, and the husband will be asked based on that placed rule if his wife has given birth at home. (WDA, Seka District)

… the husband allows his wife to use health services out of fear of the rules. (WDA, Kersa District)

… Husbands are not denying pregnant women the right to go to a health facility while they face labor; rather, they take them to the health facility. (WDA, Seka District)

##### 3.5.7.5. Belief of men that parent discussion saves life

The study highlights the regret of men for mothers who have died due to a lack of awareness and inaccessibility of maternal health services. Currently, pregnant women have access to adequate health information through discussions at home, health facilities, and various delivery methods. Husbands believe that discussions with pregnant women significantly save the lives of both women and newborn babies.

Previously, our [pregnant] mothers due to the lack of awareness and health institutions, have lost many [pregnant]mothers and sisters. But after this health center was built and started using health extension workers advice the death of mothers were stopped. (Husband, Seka district)

Most men have a strong positive belief that pregnant women should give birth in health institutions. They believe that “pregnant women should arrive at the health center with the support of people,” which is associated with the belief that “giving birth is just the same as coming back from death” (RL, Seka District).

Yes; we all [male community members] are with her once the labor begins. We go with her until her arrival at the health center. We will attentively stay there to follow her condition, whether she is being referred or not. We never go to our daily work that day. Every member of MDA, the neighbor, and the community at large should attend. We do whatever is needed; we carry if there is no ambulance. (MDA, Gomma District)

… since most husbands are preoccupied with different activities, they send their wives along with somebody else, like their kids. However, if she is getting worse from her pregnancy, he himself accompanies her. (MDA, Gomma District)

There are husbands who never want to separate from their wives, even for one day; who never want to leave them alone. Despite their awareness of the importance of maternity stay; they believe that their wives shouldn't leave them alone and stay there because they suspect many things, and as a result, they try to avoid using this service. (Husband, Gomma District)

## 4. Discussion

As part of the Safe Motherhood initiative project in Africa, the gender-based analysis from the perspective of the stakeholders from the woreda to the federal level, as well as the role of different health development armies and health professionals in MNCH, was addressed by the previous researchers (28). The current study explored the perceptions of health professionals who provided health services, the community health development armies, and religious leaders, who believed in knowing the social capital factors such as gender-based social norms, power relations, and psychosocial variations that influence the utilization of delivery and PNC services. Well-explored gender-based factors enhance the mechanisms through which men become involved in maternal healthcare services, and furthermore gives a complete picture of gender in maternal healthcare because the diverse dimension analysis provides an eminent input for policymakers and researchers (2, 5–9, 32, 33).

### 4.1. Individual and collective gender norms

Gender roles at both individual and social levels can influence the maternal healthcare-seeking behavior of pregnant women. As individuals, maternal healthcare roles often involve early appearance at health institutes during labor. Community-based women play a collective role in facilitating institutional delivery, providing psychosocial support, and reducing feelings of seclusion. They also provide care for children at home, making the home attractive and conducive during the postnatal period. This social support gives pregnant women the confidence to give birth in health institutes, as they can focus on their own health and family arrangements. A study in Bangladesh supports this, indicating that gender roles at the household and community level can improve the intention of women to seek maternal healthcare (26).

The role of a man as a husband is crucial in mobilizing others to carry pregnant women to health facilities, contributing to early intervention during labor and delivery. However, most studies on male involvement during delivery often focus on the actions of men, neglecting their motivation and intention (25). A qualitative study on African-American parents in the USA found that there is equal investment and interest in having a child, with the responsibility being willingly shared between both parents (22). This highlights the importance of the involvement of men in early healthcare to save the lives of women and fetuses.

### 4.2. Gender-based power relations

Gender-based power relations are a significant concern in maternal and child health outcomes, particularly in determining the place of delivery (26). In this study, shared decision-making was most common, often at health institutes, with involvement from families, neighbors, and health professionals. However, a qualitative study in Ethiopia found that women have no power in decision-making, even in reproductive health (28). This is because the participants were stakeholders from the district to federal level who might not clearly understand the value of societies on maternal healthcare, as discussed in the previous study. In addition, the current study findings revealed that pregnant women were the last decision-makers, particularly if they could not reach an agreement with their husbands. Women have legitimate power, and nothing prevents them from making the free choice to give birth in a health facility. Similarly, in Tanzania, women were the last decision-makers to use institutional delivery independent of the approval of their husbands (34).

The decision-making capacity of women has improved over time, with men accepting their right to make decisions about their health and fetuses. Improved systems for delivering maternal healthcare messages have significantly contributed to this. The participation of women in health development networks gives them the confidence to make decisions and receive support from their partners during delivery. In India, women make more decisions than men (35). However, the study in Ghana contradicts these findings, as community opinion leaders reveal that men control financial resources and decision-making in maternal healthcare and provide minimal support to pregnant women, as supported by health professionals (36). In another study, a man was considered the decision-maker for maternal healthcare services (37). This may be related to how the supremacy of men in the local area is unbroken. However, many health information dissemination mechanisms exist in the current study area, such as HEW and the Health Development Army (HDA), which play an important role in educating women about their maternal healthcare rights. This is congruent with the WHO recommendation encouraging male involvement in maternal healthcare, provided they respect, promote, and facilitate the choice and decision-making autonomy of women (10).

In rare cases, the husband may lead the final decision-making process, often advising and encouraging institutional delivery. This is done to prevent morbidity and mortality and to protect the pregnant woman from the burden of home delivery. This is supported by properly understanding the concepts of motivational and derived force of men, which is crucial in addressing gender inequity and improving maternal and child health outcomes (29).

### 4.3. Gender-based knowledge variation

The importance of institutional delivery has been recognized by both men and women, with most acknowledging the benefits of bleeding prevention and gaining advice. Therefore, pregnant women arrive at the maternal waiting area early without notifying anyone, sometimes even though their husbands have found them in the health institution delivery room. A study in Indonesia found that husband knowledge has a significant association with institutional delivery planning (20). The current study suggests that home delivery and men not being escorted are perceived as signs of backwardness while giving birth in health institutes is seen as a sign of modernization and rights of women. This aligns with a study in Malawi, where male involvement is seen as a means to express love (21).

The community has social norms that require the male partner to assist the pregnant woman in giving birth in a health facility; otherwise, he is considered irresponsible to his family and must accept responsibility for any unintended maternal and newborn health outcomes. This is similar to the study conducted in India, where men have the responsibility to attend institutional delivery (21). Nepal (24) is also included. However, male involvement would be reduced if they were perceived as a government rule that also stigmatized women who did not have a husband (21). Another study conducted in Malawi described the other way round, where it is believed that the involvement of men would magnify the weakness of women in the peer group and lead to the loss of independent decision-making power of the pregnant women (38). These may be associated with inadequate attitudinal change about male involvement that motivates institutional delivery (23). Men have improved knowledge about the health benefits of institutional delivery. They did not force the women to give birth at home unless they had financial insecurity. This differs from the study conducted in Afar and Kefa, where men make the majority of decisions regarding maternal healthcare. However, men tend to prevent pregnant women from attending the association meetings of women, provided that priority is given to their interests and the fear that they could have the possibility to identify their rights. This is in agreement with the study conducted in Ethiopia. The men in Afar and Kefa did not allow the pregnant women to attend meetings with the HDA due to the fear that they could have known their rights (19, 39).

### 4.4. Gender-based belief system in delivery service

The belief of women regarding the importance of maternal healthcare services has significantly changed. Most women have understood the discrepancy between receiving health services and not at the time of childbirth. This indicates that any observable evidence has the power to alter the beliefs of women. In addition, these women have experienced health issues that create an unpleasant odor while they gave birth at home, but those who gave birth in the health institutes returned home become pretty, as they have expressed to “look like brides.” This can improve the confidence of women when they return home and help them not feel ashamed when visitors come to congratulate them due to the absence of any blood-related odor. The other useful belief was that excessive bleeding might not occur when women give birth in health institutions. Even if it has occurred, health professionals can stop it. However, these contradictory beliefs remain to be observed in some women and have never been changed. It is culturally unacceptable for pregnant women to be seen naked in public, which prevents some women from receiving maternal healthcare services. Furthermore, some women believe that if their mother was born without complications at home, the same thing can happen to them, which reduces the desire of women to give birth in health facilities.

Men generally believe institutional delivery is necessary for safety, as it is associated with the belief that giving birth is like coming from death. This belief influences maternal health literacy and the decision to use health facilities during delivery. However, few beliefs of men are connected to religion, saying that everything is found in the hands of Allah and health facilities do not contribute to saving the lives of women and newborns. Understanding the subjective experiences of men, such as their beliefs and observable experiences, such as physical support and gender relations, is not considered a strategy for the involvement of men in maternal healthcare (40).

## 5. Conclusion

Gender-based roles in community members and health professionals play a crucial role in promoting maternal and newborn health. Local rules and early notification of labor by men help to prevent delays in the access of women to health institutions. Maternal health service literacy is improved through sustainable education, empowering women to make their own decisions. The final decision to give birth in a health facility is often made by women. Continuous community-level health information dissemination promotes gender-based social norms, encouraging women to seek delivery services. Proper care after delivery in health facilities is essential for repeated use. Gender-based qualitative data analysis identifies the strengths and weaknesses in maternal healthcare implementation strategies. This ensures women have the right to access necessary services and make informed decisions about their health.

## Data Availability

The original contributions presented in the study are included in the article/**Supplementary Material,** further inquiries can be directed to the corresponding author.
