# Supplementary material for: Gender-based roles, psychosocial variation, and power relations during delivery and postnatal care: a qualitative case study in rural Ethiopia
Source: Front Glob Womens Health. 2023 Oct 23;4:1155064. doi: 10.3389/fgwh.2023.1155064 (PMC10627791; doi:10.3389/fgwh.2023.1155064)
Supplement: Supplementary file 1 [file Table1.docx]

**Jimma University**

**Implementation study of Interventions to promote safe motherhood by JU-Ottawa University collaboration Project**

**Focus group discussion Interview guide for Women’s Health Development Army (WDA)**

**Time started: ___________________________**

**Focus group discussion Interview guide - WDA**

1. Do you have regular meetings on the issue of ANC, delivery and PNC? How often do you meet each other? What are the roles of health development army in promoting the health of mothers and in reducing maternal and neonatal death?
2. How do you work together with health extension workers to promote the health of mother and to reduce maternal and neonatal mortality?
3. How do you access information on maternal and child health services in the community?
4. In your community do women freely discuss pregnancy and childbirth matters? With whom? Why? If not why not?
5. What do women in your community do during their pregnancy? Any visit to health center/health post?
6. What are some serious health problems that can occur **during pregnancy**? (Which of these problems are severe? Have you ever observed or witnessed in that any pregnant woman die from those serious health problems related to pregnancy? Have you tried anything to safe life of the woman/women? What and how or why not? Can you tell more?)
7. What are some serious health problems that can occur during **labour or childbirth? (Which of those health problems are severe? Have you observed or witnessed that any woman die of those problems during childbirth?** Anything tried to safe the life of the labouring mother/mothers that you observed? By whom? Where? How? And if not why not? Can you elaborate more on this issue what should be done by the pregnant woman? Husband and family? By the community? By you? And health institutions?)
8. What are some serious health problems that can occur during after delivery within one to six weeks that could endanger the woman/women? Which of these problems are severe? Have you encountered any of these problems? What were the immediate solution/s/ done in order of actions? If no action why?)
9. Do pregnant women in your community visit to health post or health centre during pregnancy for checkups? How many times they visit if they visit? For what purpose they visit if they visit? If they did not visit why not?
10. For those mothers who visited health post/health center for Antenatal care, what services are given to them? When or at what visit for which service/s/?
11. How do you judge the function, quality of services given during ANC? Why?
12. What about the quality and coverage of maternal waiting area service provision, (functionality and quality) to impact or reduce maternal and newborn death rates? (How? Why? Or why not? What else needs to be improved?)
13. What are the factors that promotes or discourage pregnant mothers to stay at maternal waiting area? What are the solutions you suggest?
14. What are the roles of health development armies in promoting the utilization of ANC and maternal waiting area? (Were there problems? How those problems were addressed? What else be in place? By who could be provided?)
15. Do pregnant women prefer to have ANC and get delivery at Maternal waiting areas currently? Why or why not? How can the issues be addressed? Who is responsible for what?)
16. In your community how do women prepare for birth? What birth preparedness related services are found at family and community?
17. Every action needs decision. So, who decided the place of delivery (either at home or health institution or attended by relatives or medical person?). Why? Were the decisions accepted at what circumstances?).
18. How likely the condition of service provision influence Decision Making? How and why? What do you recommend to have safe delivery (or delivery attended by midwife at health center)?
19. After the birth what happened to the child and mother? What did they do? Where did they go? Any visit to health center? Why/why not? What did they do? Any visit from HEW or Community volunteer? What did they do?
20. Do you think the health problems can arise 2 days after birth? What about within 7 days, 15 days 20 days, 30 days and so on?). When it be expected no health problem associated to childbirth for the mother and newborn?
21. When do mothers start breast feeding? How long did mother’s breast feed their baby? When do mothers start additional feeding to their baby? Why and why not?
22. When does the newborn get immunized or vaccinated? By whom? If not immunized, why? (Can you mention the benefits of vaccination to the newborn? What are those benefits? Are those benefits crucial to the survival of the newborn and the life afterwards? How and why? What is/are the values held by the mothers, community members in general about the benefits of getting vaccinated to the newborn? Why and why not? Discuss more on this topic
23. Did HEWs visit your home in the last one year: How frequently they visit your home? Where you think HEWs live? What services did they give you?
24. Who supervise your efforts and actions to safe the mothers and newborn babies in your community? Are you satisfied by your actions so far? Why or why not?
25. Anything you will add or recommend before we rewind our discussions-you are well come?

**Time Ended:____________________**

***Thank you for your time and great participation***

**Back Ground Information of Interviewer**

- 1. Name of Interviewer ___________________________
  2. Sex_________________________________________
  3. Age of Interviewer_____________________________
  4. Educational level _______________________________
  5. Date of Interview ______________________Signature _________________
