# Supplementary material for: Gender-based roles, psychosocial variation, and power relations during delivery and postnatal care: a qualitative case study in rural Ethiopia
Source: Front Glob Womens Health. 2023 Oct 23;4:1155064. doi: 10.3389/fgwh.2023.1155064 (PMC10627791; doi:10.3389/fgwh.2023.1155064)
Supplement: Supplementary file 2 [file Table2.docx]

**Jimma University**

**Implementation study of Interventions to promote safe motherhood by JU-Ottawa University collaboration Project**

**Focus group discussion Interview guide for Male Development Armies (MDAs)**

**Time started: __________________________________**

**Focus group discussion Interview guide - MDA**

1. How villagers talk about pregnancy and delivery?  (Planning to have baby, safety of the pregnancy, safety of the pregnant women? How and what? If not why not?
2. Do you have regular meetings to discuss on maternal and child health? How often do you meet? What are the roles of male development army in promoting the health of mothers and in reducing maternal and newborn death?
3. Are the male development armies working together with the Health Extension Workers to promote pregnant mothers and child health? What about working together with the Women Health Development Army? What and how? If not why not?
4. Where, when and how do you access information on pregnant woman and child health services in the community?
5. In your community do women freely discuss pregnancy and childbirth matters? With whom? Why? If not why not? What about husband whose wife is getting pregnant? To whom why? If not why not?
6. What does a husband do during the time when his wife becomes pregnant? Any visit to health center/health post together? Arranging transportation means? What else?
7. Could pregnant women in your community visit to health post or health centre during pregnancy for checkups? In average how many times do you think pregnant woman need to visit while pregnant?
8. At what month of the pregnancy period do pregnant women consult the health personnel for health services in your village? For what purpose they visit? If they do not visit why not?
9. For those mothers who visited health post/health center for health checkups, what services are given to them? When or at what visit for which service/s/?
10. How do you judge, (good, bad, or else), the function, quality of services given during the pregnant women visit to health institutions? Why?
11. What about the quality and coverage of maternal waiting area service provision, (functionality and quality) towards reducing pregnant women and newborn death rates? (How? Why? Or why not? What else needs to be improved?)
12. What are the factors that promotes or discourage pregnant mothers to stay at maternal waiting area immediately close to their delivery? What are the solutions you suggest?
13. What are the roles of development army in your village in promoting the utilization of maternal and child health services utilization as well as staying inside maternal waiting areas at least for a week or two weeks? (Are there problems? How those problems were addressed? What else is need in place? By who could be provided? Why? And how?)
14. Do husband prefers his pregnant wife to stay at maternal waiting areas for a week and so and get deliver? Why or why not? Do the pregnant women prefer to stay at Maternal Waiting Areas? Why and Why not? How can be improved or addressed? Who is responsible for what?)
15. In your community how do women prepare for birth? What birth preparedness related services are found at family and community?
16. Every action needs decision. So, who decided the place of delivery (either at home or health institution or attended by relatives or medical person?). Why? Were the decisions accepted at what circumstances?).
17. How likely the condition of service provision influence Decision Making? How and why? What do you recommend to have safe delivery (or delivery attended by midwife at health center)?
18. After delivery what are the immediate care for the mother and the newborn? What did they do? Where did they go? Any visit to health center? Why/why not? What did they do? Any visit from HEW or Community volunteer? What did they do?
19. Do you think the health problems can arise 2 days after birth? What about within 7 days, 15 days 20 days, 30 days and so on?). When do we be sure no health problem that affects the newborn and the mother who gave birth due to delivery?
20. What are the major serious health problems (during pregnancy, child labor, after delivery)?
21. Was there any pregnant woman die in your village from serious health problems during pregnancy, child labor or post labor period? If yes, what was/were tried to safe life of the woman? What and how? Can you elaborate more by whom, how and when? What else do you recommend to safe lives of pregnant women on such occasions? If not tried, why not?
22. When do mothers start breast feeding? How long did mother’s breast feed their baby? When do mothers start additional feeding to their baby? Why and why not? Do husband support child breast feeding and feeding? Why or why not?
23. Do the newborn get immunized or vaccinated? When and by whom? If not why? (Can you mention the benefits of vaccination to the newborn? What are those benefits? Are those benefits crucial to the survival of the newborn and the life afterwards? How and why? What is/are the values held by the mothers, husbands, community members in general about the benefits of getting vaccinated to the newborn? Why and why not? Discuss more on this topic
24. HEWs visit your home in the last one year: How frequently they visit your home? Where you think HEWs live? What services did they give you?
25. Who supervise your efforts and actions to safe the mothers and newborn babies in your community? Are you satisfied by your actions so far? Why or why not?
26. Anything you will add or recommend before we rewind our discussions-you are well come?

- **Time ended __________________**

***Thank you for your time and great participation***

**Back Ground Information of Interviewer**

- 1. Name of Interviewer ___________________________
  2. Sex_________________________________________
  3. Age of Interviewer_____________________________
  4. Educational level _______________________________
  5. Date of Interview _________________________Signature _________________
