# Supplementary material for: Gender-based roles, psychosocial variation, and power relations during delivery and postnatal care: a qualitative case study in rural Ethiopia
Source: Front Glob Womens Health. 2023 Oct 23;4:1155064. doi: 10.3389/fgwh.2023.1155064 (PMC10627791; doi:10.3389/fgwh.2023.1155064)
Supplement: Supplementary file 4 [file Table4.docx]

**Jimma University**

**Implementation study of Interventions to promote safe motherhood by JU-Ottawa University collaboration Project**

**In-depth interview Guide (Questions) for Religious Leaders**

**Key Informants**

- **Religious Leaders**

Back Ground information of Interviewee

- Name of District_____________________
- Name of Health center______________
- Age of interviewee__________________
- Responsibility of the religious leader __________________
- **Time Started:____________________**

**In-depth interview Guide - RL**

1. What are the roles of religious leaders in promoting maternal and child health to decrease maternal and neonatal mortality and morbidity?
2. Dou you have a routine program with health development agent, male development agent and community members to discuss on the issue of maternal health? What mal –practices were changed through these discussions?
3. What are some serious health problems that can occur **during pregnancy** that could endanger the life of a pregnant woman? Which of these problems are severe? Could a woman die from [this problem] any of these problems?
4. What are some serious health problems that can occur during **labor and childbirth** that could endanger the life of a pregnant woman? Which of these problems are severe? Could a woman die from [this problem] any of these problems?
5. What are some serious health problems that can occur during **postpartum period** that could endanger the life of a pregnant woman? Which of these problems are severe? Could a woman die from [this problem] any of these problems?
6. What do women in your community do during their pregnancy? Any visit to health center/health post? How many times in total pregnant women should receive antenatal care during pregnancy?
7. What are the services given to pregnant women during antenatal care?
8. How do you understand the function, quality of services and impact of maternal waiting area in reducing maternal and neonatal mortality?
9. What are the factors that discourage pregnant mothers not to stay at maternal waiting area before immediately close to their delivery? What are the solutions you suggest?

1. What are the leading reasons why women not prefer to follow antenatal care services? What are the possible solution do you suggest to curb such problem?
2. In your community how do women prepare for birth? What birth preparedness related services are found in your community?
3. In your locality where do women prefer to give birth and to be assisted by? Why?
4. Top reasons why women prefer to give birth in a home rather than elsewhere?
5. In your community who will make final decision where the women give birth and birth assistance? why?
6. What is the nature & extent of husband`s involvement in decisions on the use of maternal and child health services? What happens if husbands disapprove seeking the care?
7. Why all women do not seek delivery care at health facilities?
8. Do you think the health problems can arise 2 days after birth? What are the health problems that can happen during that period? do you think it is necessary? do women get check up after birth for their health? why**?**
9. Do pregnant women in your community seek care after their delivery? Where did they prefer to go? Why?
10. Could you name some types of basic care that can be provided to a newborn baby immediately after birth?
11. In your community do women freely discuss pregnancy and childbirth matters? with whom? Why?
12. What are problems in your community that hinder pregnant women from getting health services childbirth/labor, and post partum period?
13. What preparations are found in your community for emergencies? do people make advance preparations? What barriers those hinder from such preparations?
14. What are the roles of health development army in promoting the health of mothers and in reducing maternal and neonatal death?
15. How do you do with health extension workers to promote the health of mother and to reduce maternal and neonatal mortality?

***Time Ended:____________________***

***Thank you for your time and great participation***

**Back Ground Information of Interviewer**

- 1. Name of Interviewer ___________________________
  2. Sex_________________________________________
  3. Age of Interviewer_____________________________
  4. Educational level _______________________________
  5. Date of Interview _____________________Signature _________________
