# Supplementary material for: Gender-based roles, psychosocial variation, and power relations during delivery and postnatal care: a qualitative case study in rural Ethiopia
Source: Front Glob Womens Health. 2023 Oct 23;4:1155064. doi: 10.3389/fgwh.2023.1155064 (PMC10627791; doi:10.3389/fgwh.2023.1155064)
Supplement: Supplementary file 5 [file Table5.docx]

**Annex I: Additional files IDI Guides]**

**Jimma University**

**Implementation study of Interventions to promote safe motherhood by JU-Ottawa University collaboration Project**

**In-Depth Interview guide for PHCUD and Midwifery Nurse**

**Key Informants**

- **PHCUD and Midwifery Nurse**

Back Ground information of Interviewee

- Name of District_____________________
- Name of Health center and Health post______________
- Age of interviewee__________________
- Responsibility of the HEW __________________
- **Time started __________________**

**In-depth interview Guide – PHCUD and Midwifery Nurse**

1. How do you break down your time for maternal health services and other health extension programs? How much of your work week is for maternal and child health program?
2. Give a detailed list of maternal and child health services you provide for the community?
3. What do you do to encourage members of your community so that they participate in maternal and child related activities, education or planning?
4. What are some serious health problems that can occur **during pregnancy** that could endanger the life of a pregnant woman? Which of these problems are severe? Could a woman die from [this problem] any of these problems?
5. What are some serious health problems that can occur during **labour and childbirth** that could endanger the life of a pregnant woman? Which of these problems are severe? Could a woman die from [this problem] any of these problems?
6. What are some serious health problems that can occur during **postpartum period** that could endanger the life of a pregnant woman? Which of these problems are severe? Could a woman die from [this problem] any of these problems?
7. How many ANC attendants could you face each month on average?
8. What are the services given to pregnant women during antenatal care visits?
9. What was the level of ANC 1 to ANC 4 dropout rate? What are the reasons for high dropout rate?
10. How do you understand the function, quality of services and impact of maternal waiting area in reducing maternal and neonatal mortality?
11. What are the factors that discourage pregnant mothers not to stay at maternal waiting area before immediately close to their delivery? What are the solutions you suggest?
12. What are the roles of health extension workers to increase the uptake of Maternal Waiting Area?
13. What are the leading reasons why women not prefer to follow antenatal care services? What are the possible solution do you suggest to curb such problem?
14. In your community how do women prepare for birth? What birth preparedness related services are found in your community?
15. In your locality where do women prefer to give birth and to be assisted by? Why?
16. Top reasons why women prefer to give birth in a home rather than elsewhere?
17. Why all women do not seek delivery care at health facilities?
18. Do you think the health problems can arise after birth? What are the health problems that can happen during that period? do you think it is necessary? Do women get check up after birth for their health? Why**?**
19. Do pregnant women in your community seek care after their delivery? Where did they prefer to go? Why?
20. Could you name some types of basic care that can be provided to a newborn baby immediately after birth?
21. What are problems in your community that hinder pregnant women from getting health services childbirth/labor, and post partum period?
22. How do you involve men during household visit or health education concerning maternal and child health?
23. How do you understand preparation for birth? What practices are common in your community? Who is responsible for saving money? How do women get financial support?
24. How do you work together with health development army, opinion leaders and model households to promote the health of mother and to reduce maternal and neonatal mortality?
25. When did you refer pregnant women for ANC, Delivery services and PNC to upper level of health level of health system? Where? How?
26. What did you do after they return to the community?
27. How often health center offices visited your community pertaining to maternal and child health services? What they do? What does the guideline say about the visit
28. How do you get feedback from health center officers or district health offices regarding maternal and child health services? Does the feedback have values?
29. Do you have routine meeting with health development armies? In what ways community residents participate in maternal and child health activities?

- **Time ended __________________**

***Thank you for your time and great participation***

**Back Ground Information of Interviewer**

- 1. Name of Interviewer ___________________________
  2. Sex_________________________________________
  3. Age of Interviewer_____________________________
  4. Educational level _______________________________
  5. Date of Interview ________________________Signature _________________
